# Spinopelvic sagittal balance: what does the radiologist need to know?

**DOI:** 10.1590/0100-3984.2019.0048

**Published:** 2020

**Authors:** Leonor Garbin Savarese, Rafael Menezes-Reis, Gustavo Perazzoli Bonugli, Carlos Fernando Pereira da Silva Herrero, Helton Luiz Aparecido Defino, Marcello Henrique Nogueira-Barbosa

**Affiliations:** 1 Hospital das Clínicas da Faculdade de Medicina de Ribeirão Preto da Universidade de São Paulo (HCFMRP-USP), Ribeirão Preto, SP, Brazil.

**Keywords:** Spine, Pelvis, Radiography, Lordosis/diagnostic imaging, Kyphosis/diagnostic imaging, Spinal curvatures/diagnostic imaging, Postural balance, Coluna, Pelve, Radiografia, Cifose/diagnóstico por imagem, Lordose/diagnóstico por imagem, Curvaturas da coluna vertebral/diagnóstico por imagem, Equilíbrio postural

## Abstract

Sagittal balance describes the optimal alignment of the spine in the sagittal plane, resulting from the interaction between the spine and lower limbs, via the pelvis. Understanding sagittal balance has gained importance, especially in the last decade, because sagittal imbalance correlates directly with disability and pain. Diseases that alter that balance cause sagittal malalignment and may trigger compensatory mechanisms. Certain radiographic parameters have been shown to be clinically relevant and to correlate with clinical scores in the evaluation of spinopelvic alignment. This article aims to provide a comprehensive review of the literature on the spinopelvic parameters that are most relevant in clinical practice, as well as to describe compensatory mechanisms of the pelvis and lower limbs.

## Abstract (pt)

O equilíbrio sagital descreve o alinhamento ideal da coluna vertebral no plano sagital, sendo resultante da interação entre a coluna e os membros inferiores, por meio da bacia. O entendimento do equilíbrio sagital ganhou importância principalmente na última década, uma vez que o desequilíbrio sagital se correlaciona diretamente com incapacidade e dor. Doenças que alterem este equilíbrio podem causar mal alinhamento sagital e desencadear mecanismos compensatórios, os quais tentam restaurar a postura ereta. A literatura relata parâmetros radiográficos clinicamente relevantes, que possuem relação estabelecida com escores clínicos, para avaliar o alinhamento espinopélvico. Este artigo tem como objetivo fornecer uma ampla revisão da literatura sobre os parâmetros espinopélvicos mais relevantes na prática clínica, e descrever os mecanismos compensatórios da pelve e membros inferiores.

## INTRODUCTION

The human spine presents high biomechanical complexity, allowing bipedalism and resulting in the development of S-shaped curvatures of the spine^(1)^. The relationship between the pelvis and the spine is a direct effect of bipedalism. Adopting a vertical posture resulted in the expansion and the verticalization of the pelvis, which has led to the emergence of sagittal curves in the spine-including cervical lordosis, thoracic kyphosis, and lumbar lordosis-as well as to modifications in the tissues that stabilize the spine^(2)^.

Spinal deformity in the sagittal plane has been considered one of the main causes of disability, with a significant impact on health, and reputed by some authors as equivalent to diseases such as cancer, diabetes, and heart disease^(3-7)^. As the western population has aged, adult spine deformity has become more prevalent, with estimated rates of up to 60% in the elderly population of the United States^(8)^. The understanding of sagittal balance has gained importance in the last decade, with evidence that an imbalance is directly correlated with disability and pain^(3)^.

Introduced in the mid-1980s, the concept of sagittal balance has been widely used in the evaluation and management of disorders of the spine, with a growing interest in the study of spinopelvic parameters over the last three decades^(3-9)^. Continuing the work of During et al.^(10)^ and Duval-Beaupère et al.^(11)^, several authors have emphasized the importance of sagittal balance in diseases of the spine^(12,13)^. Roussouly et al.^(14)^ played a fundamental role in promoting the concept, creating a classification of the asymptomatic population based on spinopelvic parameters. Other studies involving asymptomatic patients showed correlations between spinopelvic parameters and sagittal curvatures of the spine. One study, conducted by Glassman et al.^(3)^, became a classic reference that established the sagittal vertical axis as the primary measure of sagittal deformity.

The present article aims to provide a comprehensive review of the literature on the spinopelvic parameters that are most relevant in clinical practice, as well as to describe the compensatory mechanisms of the pelvis and lower limbs.

## THE EXAMINATION TECHNIQUE

**Figure 1:**
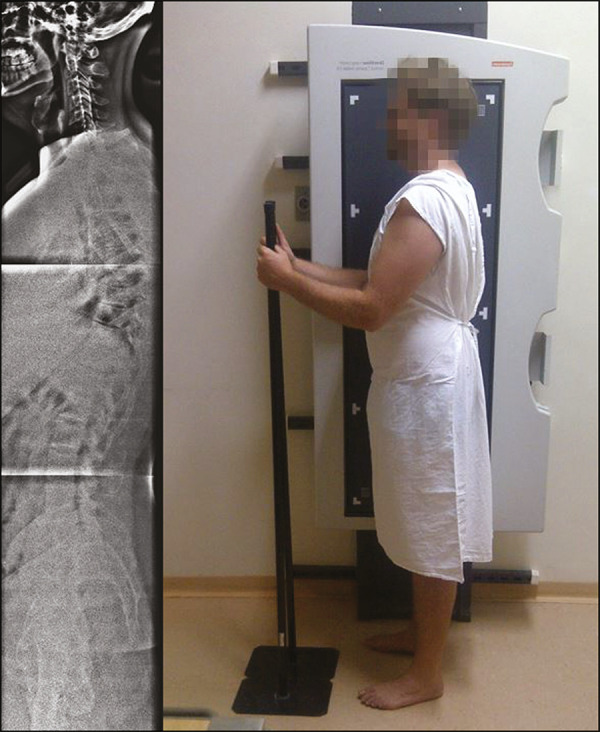
Panoramic X-ray acquired in the sagittal plane (on the left) and patient positioning for its acquisition (on the right).

For the radiographic evaluation of the sagittal plane, it is necessary to acquire a panoramic lateral view of the spine that enables evaluation all the way from the craniovertebral junction to the femoral heads, which would need to be included within the field of vision and with visible contours. There are different ways of positioning the patient for a panoramic spine radiography. Based on the study of Marks et al.^(15)^, the patient should be standing, with the upper limbs resting on a support, the shoulders at 30° forward flexion, and the elbows slightly flexed (Figure 1). That position is most comparable to the functional standing position, in which both arms hang at the sides, and does not significantly alter the sagittal alignment^(15)^.

## SPINOPELVIC PARAMETERS

### Pelvic parameters

The most commonly used pelvic parameters are the pelvic tilt, the sacral slope, and the pelvic incidence.

#### Pelvic tilt

**Figure 2:**
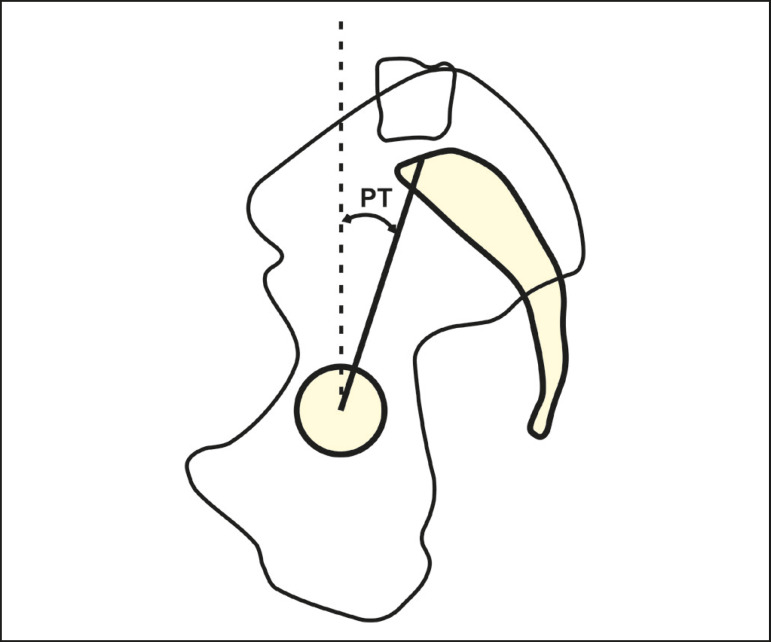
Schematic representation of the pelvic tilt (PT), which is the angle formed by a vertical line originating from the midpoint of the femoral head and a line running from the midpoint of the femoral head to the midpoint of the upper endplate of S1.

The pelvic tilt is the angle formed between the vertical and a line connecting the midpoint of the femoral heads to the midpoint of the upper endplate of S1 (Figure 2). In simple terms, this angle describes the rotational orientation of the pelvis around the femoral heads. It varies between 5° and 30°, with an average of 12°^(5,16,17)^. In most cases, the two femoral heads do not overlap perfectly in the lateral view. In such cases, we should use the geometric center of the femoral heads, which is the midpoint of the line that connects the geometric centers of the femoral heads. Lafage et al.^(6)^ demonstrated that a greater pelvic tilt correlates with worse quality of life and poorer health status.

#### Sacral slope

**Figure 3:**
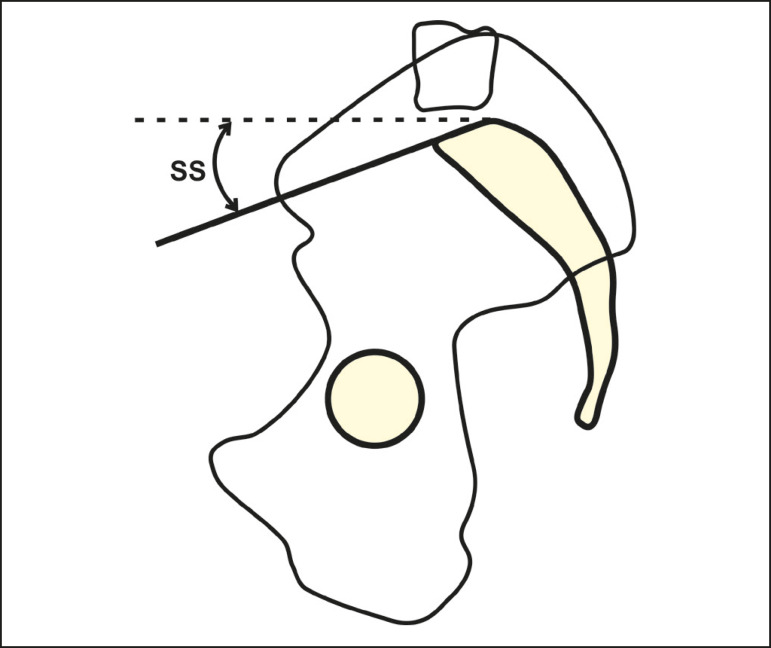
Schematic representation of the sacral slope (SS) angle, which is the angle between the upper endplate of S1 and a horizontal line.

The sacral slope is the angle formed between the horizontal and the upper endplate of S1 (Figure 3). It varies between 20° and 65°, with an average of 40°^(16)^, and has a direct correlation with lumbar lordosis.

#### Pelvic incidence

**Figure 4:**
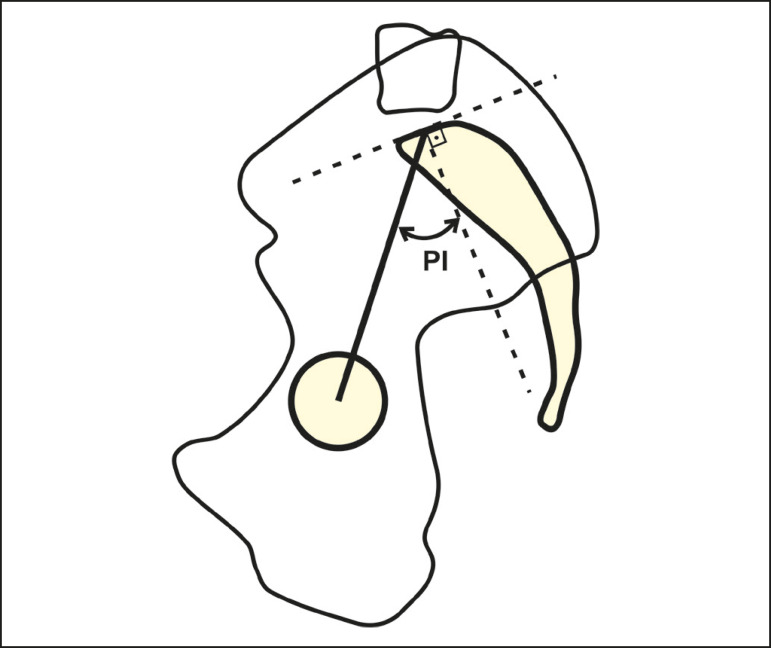
Schematic representation of the pelvic incidence (PI), which is the angle between a line perpendicular to the upper endplate of S1 and a line connecting the midpoint of that endplate with the midpoint of the femoral head.

The pelvic incidence is the angle between a perpendicular line to the upper endplate of S1 and the line connecting the midpoint of that endplate with the midpoint of the femoral rotation (Figure 4).

The pelvic incidence is a morphological parameter that is independent of the spatial orientation of the pelvis and is considered specific for each individual^(5)^. The pelvic incidence value grows progressively during adolescence and becomes constant in adulthood. It is directly related to the value of the lumbar lordosis and ranges from 34° to 84°, with an average of 52°^(5,16,17)^. There is a geometric relationship between those parameters: pelvic incidence = pelvic tilt + sacral slope. It is important to understand that the pelvic incidence is a descriptive parameter of pelvic morphology and not of its orientation, therefore, its angular value is not affected by postural changes. In contrast, the pelvic tilt and sacral slope vary and are useful for characterizing the spatial orientation of the pelvis. The pelvic incidence is related to the ability that each individual has to compensate for sagittal imbalance. A high pelvic incidence accompanied by a high sacral slope and a low pelvic tilt indicates a greater capacity for spinopelvic compensation. In contrast, a low pelvic incidence indicates less capacity for spinopelvic compensation.

**Figure 5:**
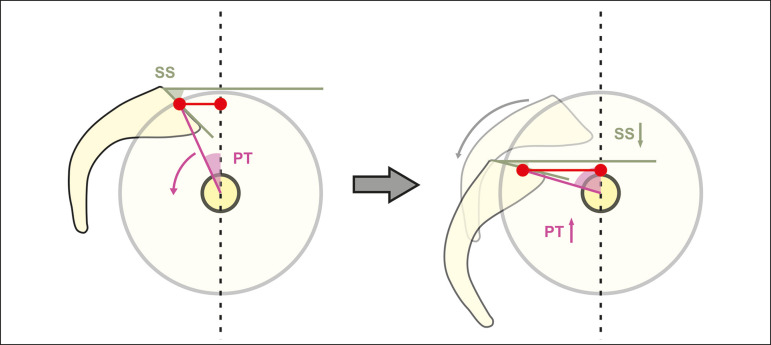
Schematic representation of pelvic retroversion. By bringing the upper endplate of S1 closer to the axis of the femoral heads and increasing the sacrofemoral distance, this mechanism compensates for the anterior shift in the center of gravity. Adapted from Barrey et al.^(9)^. SS, sacral slope; PT, pelvic tilt.

The rotation of the pelvis (a change in the pelvic tilt) causes combined movements of rotation and shifting of the S1 vertebra. These dislocations result in changes in the sacral slope and modify the relative position of the lumbosacral junction in relation to the hips (Figure 5). Therefore, increasing the pelvic tilt (extending the hips) is a posterior shift that will decrease the sacral slope through verticalization of the S1. Conversely, decreasing the pelvic tilt (flexing the hips) results in an anterior shift that will increase the sacral slope through horizontalization of the S1^(17-20)^.

### Vertebral parameters

The most commonly used vertebral parameters are thoracic kyphosis, lumbar lordosis, the sagittal vertical axis, the difference between the pelvic incidence and lumbar lordosis (PI−LL), the T1 pelvic angle, global tilt, the spinopelvic angle, and the spinosacral angle.

#### Thoracic kyphosis

**Figure 6:**
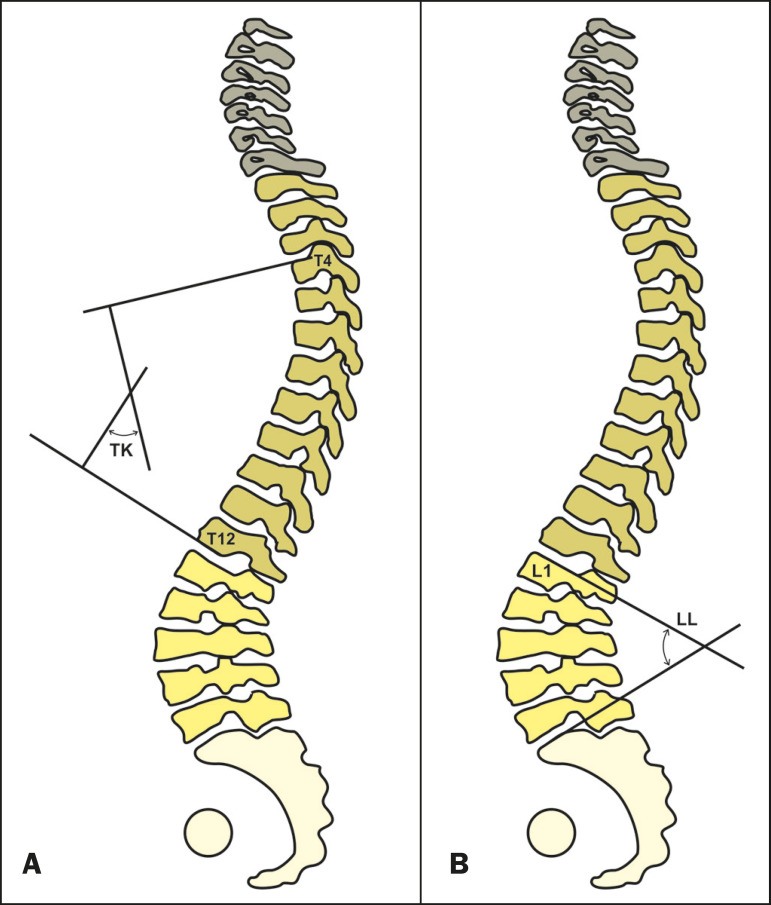
**A:** Schematic representation of the thoracic kyphosis (TK) angle, which is the measurement of the Cobb angle between the lower endplate of T12 and the upper endplate of T4. **B:** Schematic representation of the lumbar lordosis (LL) angle, which is the measurement of the Cobb angle between the upper endplate of S1 and the upper endplate of L1.

The identification of thoracic kyphosis is based on the measurement of the Cobb angle between the lower endplate of T12 and the upper endplate of T1^(21)^. However, the position of the shoulder on an X-ray can overlap the image and hinder the localization of T1. To avoid this problem, some authors suggest using the upper endplate of T4 to measure thoracic kyphosis^(22)^. As depicted in Figure 6A, the Cobb angle between T4 and T12 ranges from 20° to 50° in normal individuals^(23)^.

#### Lumbar lordosis

The identification of lumbar lordosis is based on the measurement of the Cobb angle between the upper endplate of S1 and the upper endplate of L1^(6,24,25)^, as shown in Figure 6B. It normally ranges between 30° and 79°^(18,19,26)^. Two recent studies have used statistical calculations to predict the ideal lumbar lordosis using formulas based on pelvic incidence. With a multilinear regression analysis, Legaye et al.^(27)^ proposed a formula for the prediction of the ideal lumbar lordosis: lumbar lordosis = −[(pelvic incidence × 0.5481 + 12.7) × 1.087 + 21.61]. Subsequently, Schwab et al.^(22)^ proposed a simpler approach, and estimated the ideal lumbar lordosis based on an asymptomatic adult population (75 individuals; mean age of 48 ± 18 years): lumbar lordosis = pelvic incidence + 9° (± 9).

#### Sagittal vertical axis

**Figure 7:**
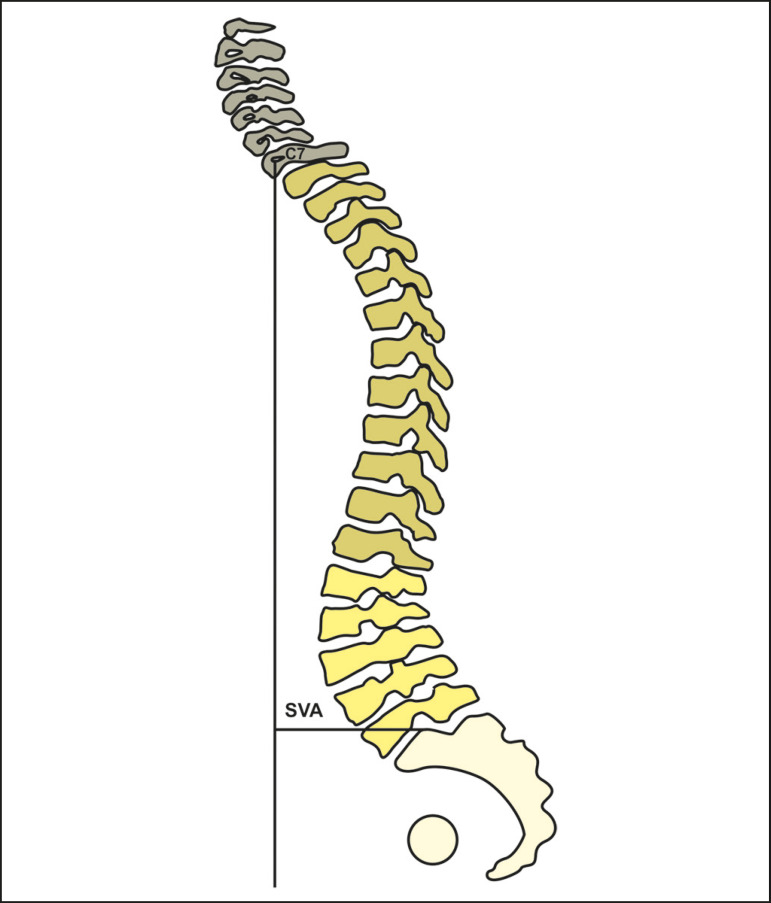
Schematic representation of the sagittal vertical axis (SVA), which is the horizontal distance between the C7 plumbline and the posterior edge of the upper endplate of S1.

The sagittal vertical axis is the measurement of the horizontal distance between the C7 plumbline and the vertical line passing through the upper posterior edge of S1 (Figure 7). This serves to document the location of the head in relation to the center of gravity (C7 deviation in relation to the sacral promontory). Jackson et al.^(18)^ reported values in asymptomatic adults with a mean sagittal vertical axis deviation of 0.5 ± 2.5 cm. Glassman et al.^(3)^ showed that, among 352 patients with positive sagittal alignment, a high sagittal vertical axis correlated with pain and worse scores for health and quality of life.

#### PI−LL

The PI−LL is often used as a descriptive parameter for the spine alignment^(6,18)^. Schwab et al.^(28)^ found that the PI−LL correlated significantly with the pelvic tilt (r = 0.844; *p* < 0.001) and the sagittal vertical axis (r = 0.685; *p* < 0.001). It has been suggested that a PI−LL below 10° indicates a malalignment. Because the lumbar lordosis value must adapt to the pelvic morphology (evaluated by the pelvic incidence), a lack of correspondence between the two values would represent a condition in which the patient could not find a spinopelvic organization in accordance with their pelvic anatomy. The PI−LL showed a correlation with questionnaires related to health and quality of life, simultaneously proving to be a valuable tool for the intraoperative planning of correction of flat back syndrome^(4,6,7,29)^, being used as the basis for determining the target correction in surgical treatment of sagittal malalignment.

#### T1 pelvic angle and global tilt

Recently, the T1 pelvic angle and the global tilt were proposed as new spinopelvic parameters that represent both spinal inclination and pelvic retroversion. These parameters do not change with postural compensation^(4,30)^.

**Figure 8:**
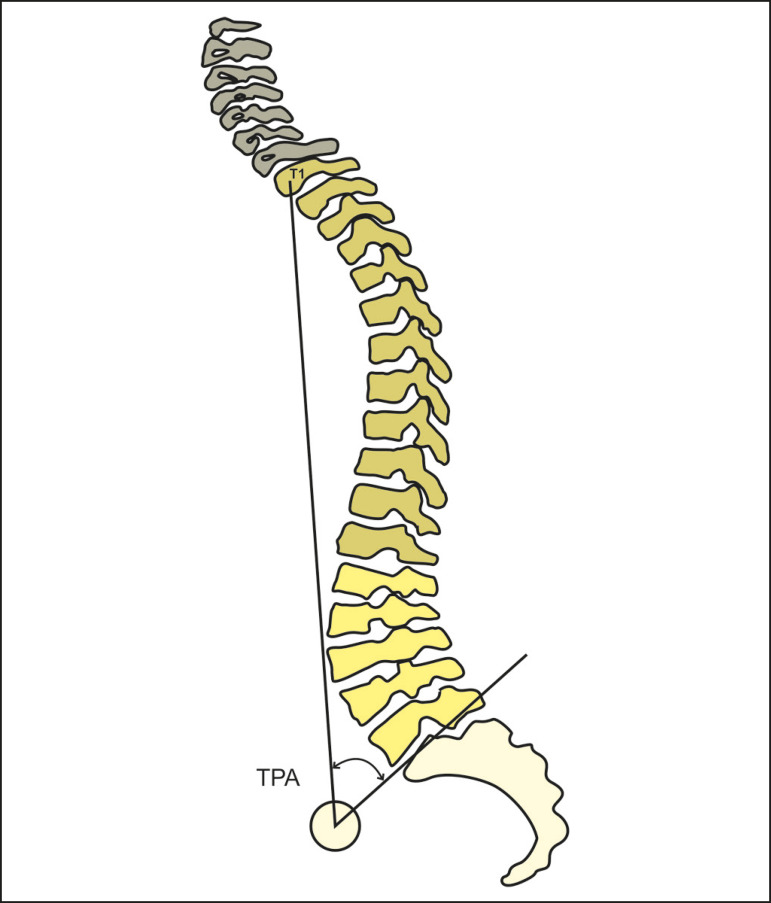
Schematic representation of the T1 pelvic angle (TPA), which is the angle between the line running from the midpoint of the vertebral body of T1 to the geometric midpoint of the femoral heads and the line running from the geometric midpoint of the femoral heads to the midpoint of the upper endplate of S1.

The T1 pelvic angle is the angle between the line running from the geometric midpoint of the femoral heads to the midpoint of the vertebral body of T1 and that running from the geometric midpoint of the femoral heads to the midpoint of the upper endplate of S1 (Figure 8). It corresponds to the sum of the T1 spinopelvic inclination and the pelvic tilt.

In the study conducted by Protopsaltis et al.^(4)^, the T1 pelvic angle was found to correlate with the sagittal vertical axis (r = 0.837), the PI−LL (r = 0.889), and the pelvic tilt (r = 0.933), as well as with questionnaires related to the health and quality of life of patients with adult spinal deformity. The authors suggested that it is a useful tool in the preoperative planning for such patients, the target T1 pelvic angle being < 14°.

**Figure 9:**
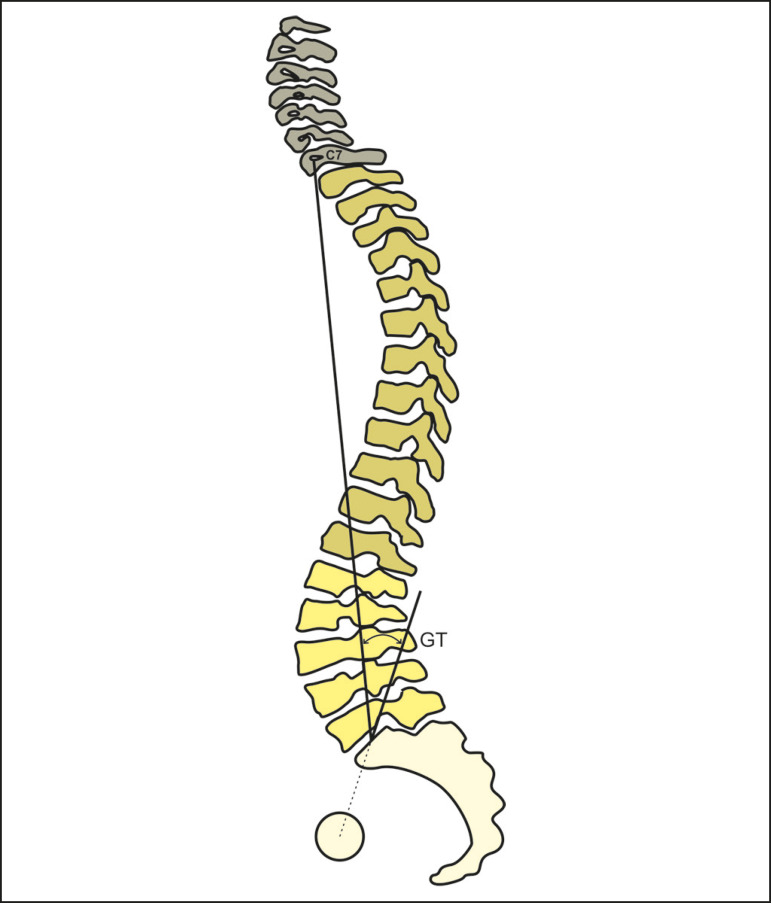
Schematic representation of the global tilt (GT), which is the angle between the line running from the midpoint of the vertebral body of C7 to the midpoint of the upper endplate of S1 and the line running from the midpoint of the upper endplate of S1 to the geometric midpoint of the femoral heads.

The global tilt is the angle between the line running from the midpoint of the upper endplate of S1 to the midpoint of the vertebral body of C7 and that running from the geometric midpoint of the femoral heads to the midpoint of the upper endplate of S1 (Figure 9).

The T1 pelvic angle and the global tilt combine the trunk anteversion and pelvic retroversion as a parameter to evaluate the overall spinal deformity. Recently, Banno et al.^(31)^ described reference values for the global tilt in elderly individuals. In that study, the global tilt seemed to be related to age-the mean global tilt being 15.4 ± 8.7° for the patients between 50 and 59 years of age and 30.8 ± 14.8° for the patients ≥ 80 years of age-and gender-the mean global tilt being 26.0° for women and 18.8° for men-showing a high correlation with the pelvic tilt (r = 0.914) and the sagittal vertical axis (r = 0.751). The authors proposed a cutoff global tilt value of 33.7° for an Oswestry Disability Index of > 40. These results underscore the fact that the interpretation of the global tilt values, like that of other sagittal parameters, should be made according to the general context of patients with deformities of the spine.

#### Spinopelvic angle

**Figure 10:**
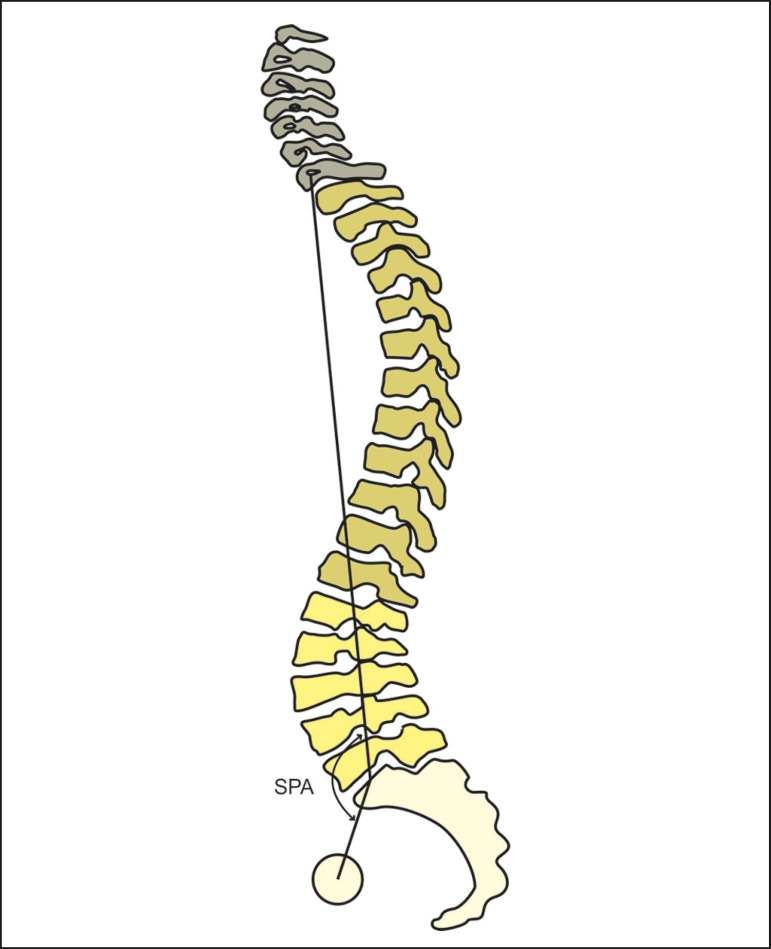
Schematic representation of spinopelvic angle (SPA), which is the angle between the line running from the midpoint of C7 to the midpoint of the upper endplate of S1 and the line running from the midpoint of the upper endplate of S1 to the geometric midpoint of the femoral heads.

The spinopelvic angle is the angle between the line running from the midpoint of C7 to the midpoint of the upper endplate of S1 and that running from the midpoint of the upper endplate of S1 to the geometric midpoint of the femoral heads (Figure 10). This angle evaluates the overall spinopelvic alignment, taking into consideration the retroversion of the pelvis and the anteversion of the trunk.

#### Spinosacral angle

**Figure 11:**
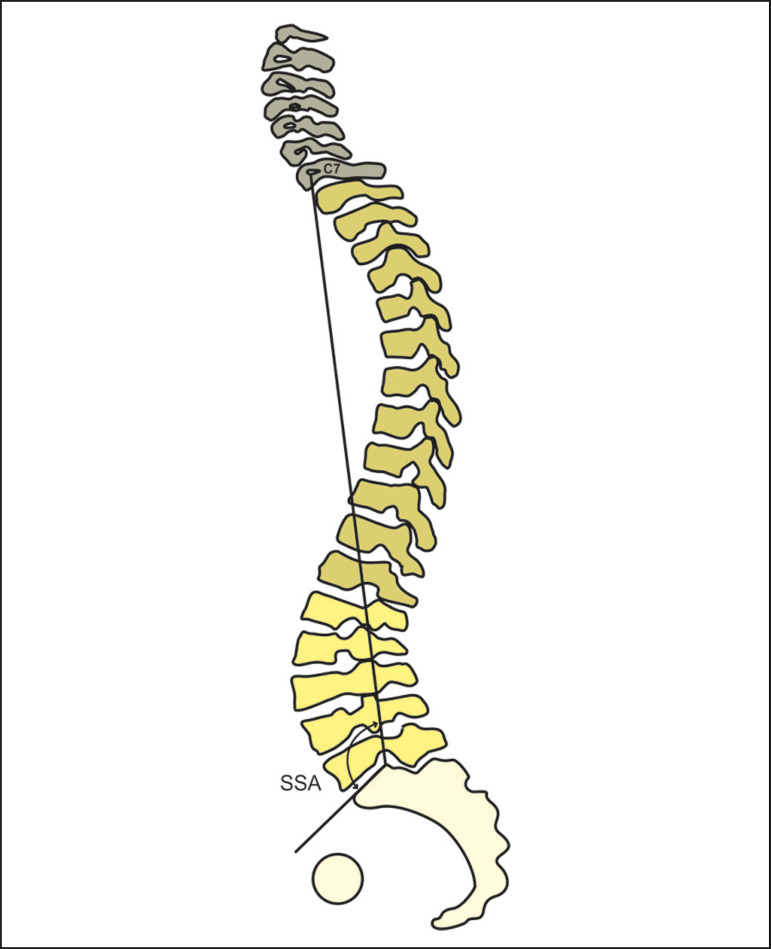
Schematic representation of spinosacral angle (SSA), which is the angle formed by the line that runs from the midpoint of C7 to the midpoint of the upper endplate of S1 and the surface of that same endplate.

As depicted in Figure 11, the spinosacral angle is the angle between the line running from the midpoint of C7 to the midpoint of the upper endplate of S1 and the surface of that endplate; it was conceived as a means of quantifying the overall kyphosis of the spine as a whole. The average value of the spinosacral angle in asymptomatic individuals is 130.4 ± 8.1°^(32)^. In a study involving a healthy population^(19)^, the spinosacral angle was found to correlate strongly with the sacral slope (r = 0.914; *p* < 0.001) and the lumbar lordosis (r = 0.889; *p* < 0.001). Other studies have shown that the spinosacral angle is lower in the presence of diseases such as degenerative kyphosis^(33)^, rheumatoid arthritis^(34)^, and discogenic lumbar pain^(35)^. In addition, the spinosacral angle is associated with the presence of pain and functional alterations^(34)^.

### Relationships between spinopelvic parameters and the Roussouly classification

The recognition of the relationship between pelvic morphology, represented by the pelvic incidence, and the lumbar lordosis improved understanding of malalignment of the spine in the sagittal plane^(5)^. The pelvic incidence value is an individual anatomical characteristic and corresponds to the “thickness” of the pelvis. More than two decades ago, Legaye et al.^(5)^ and Duval-Beaupère et al.^(11)^ postulated that a high pelvic incidence is associated with a high sacral slope and pronounced lumbar lordosis, and that a low pelvic incidence is associated with a lower sacral slope and subtle lumbar lordosis, leading to the basic concept of an “economic standing position”^(36)^.

**Figure 12:**
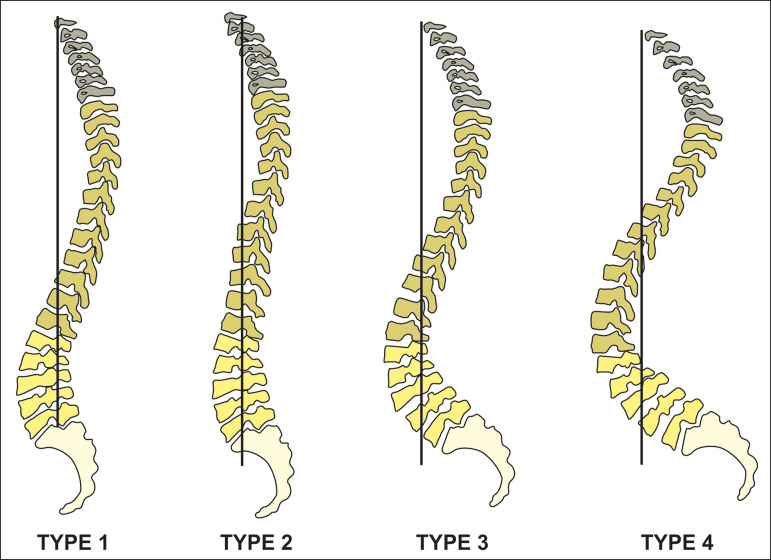
Postural types according to the Roussouly classification.

Based on the concept that the morphology and spatial orientation of the pelvis determine the organization of the spine and its curvatures, Roussouly et al.^(14)^ created a classification in the asymptomatic population based on their observation of a strong correlation between the lumbar lordosis and the sacral slope. After observing that there are characteristic sagittal profiles that vary depending on the spatial orientation of the pelvis, the authors described four patterns of sagittal alignment variation, as depicted in Figure 12. Roussouly type 1 is characterized by an sacral slope value of less than 35° and a low pelvic incidence. The lower arch of the lordosis is minimal, with a short lumbar lordosis and a long thoracolumbar kyphosis. Type 2 is characterized by an sacral slope value of less than 35° and a low pelvic incidence, the lower arch of the lordosis being relatively flat. The entire spine is relatively hypolordotic and hypokyphotic. Type 3 is characterized by an sacral slope value between 35° and 45°, greater prominence of the lower arch of the lordosis, and better balance of the spine, the thoracic kyphosis and lumbar lordosis being in harmony. Type 4 is characterized by an sacral slope value greater than 45°, accompanied by a high pelvic incidence and prominence of the lower arch of the lordosis.

### Compensatory mechanisms

There is no single pattern of sagittal balance in the normal population^(20)^. It is essential to have a very high congruence between the pelvis and the spine to achieve an economic posture with the center of gravity in a given physiological position^(20,37)^. The ultimate goal of spinopelvic balance is to keep the center of gravity near the center of the hips^(19)^. The interaction between the spine and the pelvis is an important key point in the analysis of spinal deformities. This interaction is further modulated by the compensatory mechanisms to maintain a permanent alignment of the horizontal position and horizontal gaze^(36,38,39)^. The sagittal imbalance is basically triggered by a maladjustment between the pelvic incidence and lumbar lordosis, due to disc degeneration, trauma, or iatrogenic fusion. The reduction of the lumbar lordosis results in an anterior flexion of the trunk, characterized by an increase in the sagittal vertical axis, giving rise to a curved posture and a downward tilt of the head, with the consequent inability to see above the horizon. When this imbalance occurs, compensatory mechanisms are activated in order to restore a compensated balance. The compensation begins at the level of the spine with modifications of the curvatures, reduction of the kyphosis, and lumbar hyperextension. If this compensation is insufficient, the patient tilts the pelvis backwards and extends the hips, increasing the value of pelvic tilt and reducing the sagittal vertical axis. In addition, some lumbar segments may present hyperextension or retrolisthesis, thus increasing the risk of stenosis of the vertebral canal. Finally, when all compensatory mechanisms have been exhausted, the lower limbs may help restore a horizontal gaze by flexion of the knees^(38,40)^. The flexion of the knees affects the magnitude of the sagittal vertical axis allowing greater pelvic retroversion to retract the head over the hips and ankles, effectively reducing the sagittal vertical axis^(36,38,39)^.

## DISCUSSION

Studies have confirmed that patients with lumbar degenerative disease are characterized by anterior sagittal imbalance, loss of lumbar lordosis, and increased pelvic tilt^(41,42)^. The sagittal vertical axis and pelvic tilt are spinopelvic parameters that reflect the severity of the adult spine deformity, however, there are some points to be considered. First, the measurement of the sagittal vertical axis can be diminished by postural compensation mechanisms such as pelvic retroversion. Therefore, a high pelvic tilt can “hide” a greater spinal deformity when only the sagittal vertical axis is being considered. The sagittal vertical axis and pelvic tilt are inter-related in the sense that the magnitude of one affects the other. Lafage et al.^(6)^ suggested that the pelvic tilt should be considered in conjunction with the sagittal vertical axis to identify patients with spinal deformity in the sagittal plane without a high sagittal vertical axis due to pelvic compensation. It is worth mentioning that a successful realignment plan should not only restore the spinopelvic relationship but also zero out the compensatory mechanisms, which are energy drains and affect patient quality of life. Therefore, the sagittal vertical axis of the patient should not be considered in isolation when evaluating the sagittal plane. In contrast, the T1 pelvic angle and the global tilt have many advantages for the assessment of global alignment because they consider the retroversion of the pelvis and anteversion of the trunk, as well as because they are not affected by postural or radiographic calibrations^(4,38)^. In addition, the T1 pelvic angle and global tilt both correlate strongly with the sagittal vertical axis, pelvic tilt, and PI−LL^(31)^. Based on these concepts, we suggest the use of the global tilt, spinopelvic angle, and T1 pelvic angle parameters in clinical practice, because they are less prone to postural variation than the sagittal vertical axis.

Roussouly et al.^(40)^ described spinopelvic relationships by means of the spinopelvic angle. The spinopelvic angle is similar in concept to the T1 pelvic angle and global tilt, because it also assesses the global spinopelvic alignment, taking into consideration the retroversion of the pelvis and the anteversion of the trunk. However, the spinopelvic angle decreases as the deformity increases, and the pathological values of spinopelvic angle that correlate with health-related quality of life have yet to be established. In reality, global tilt is a modified version of the spinopelvic angle described by Roussouly. From a geometric point of view, this angle corresponds to the following formula: spinopelvic angle = 180 − global tilt. The spinopelvic angle and global tilt are in fact supplementary angles.

The compensatory mechanisms of the pelvis can be quantified by the pelvic tilt, an angle proposed by Duval-Beaupère et al.^(11)^, which was subsequently correlated with health-related quality of life scores by Lafage et al.^(6)^. Theoretically, individuals with a low pelvic incidence would have a greater anterior acetabular anteversion with greater hip extension and, as a result, less capacity to adapt to sagittal malalignment^(43)^. Lafage et al.^(6)^ demonstrated that, when they categorized patients with deformity into four groups by pelvic tilt (higher and lower) and sagittal vertical axis (higher and lower), the group with the worst score on the Oswestry Disability Index showed higher sagittal vertical axis values and lower pelvic incidence values, suggesting that patients with greater deformities and less ability to compensate with pelvic retroversion would have higher levels of disability.

Roussouly et al.^(21)^ performed a biomechanical analysis of the spinopelvic organization, categorized by types, which has been said to describe the degenerative evolution of the spine. Roussouly et al.^(14)^ suggested that patients with symptomatic disc disease are most commonly classified as types 1 or 2, while the stenosis of the vertebral canal is usually associated with type 4, whereas type 3 is rarely seen in patients with disorders of the spine. Chaléat-Valayer et al.^(44)^ found a higher proportion of patients with chronic lumbar pain whose sacral slope, pelvic incidence, and lumbar lordosis were all lower than the control individuals, suggesting a relationship between this specific pattern (Roussouly type 2) and the presence of lumbar pain. Menezes-Reis et al.^(45)^ found that Roussouly type 2 was associated with disc degeneration in the L4-L5 segment in asymptomatic individuals.

More recently, the first description of the sagittal alignment of the spine with degenerative disease based on its shape was published^(46)^, identifying 11 types. The newly proposed classification includes the four classic types of Roussouly along with seven other types found in degenerated spines: anteverted type 3; anteverted type 4; false type 2; false type 2 with thoracic kyphosis; false type 3, lumbar kyphosis; and global kyphosis. That study proposes a possible explanation for the fact that the classical subtypes seen in healthy individuals have evolved into pathological types.

Research in asymptomatic individuals has shown a correlation between spinopelvic parameters and paravertebral muscle volume, although none of those parameters have been found to correlate with fatty infiltration of muscle^(47)^. The volume of the psoas muscle showed a positive correlation with the magnitude of the thoracic kyphosis, lumbar lordosis, and sagittal vertical axis^(47)^.

Previous studies have found that patients with lumbar pain have a decreased sacral slope, an increased pelvic tilt, and a reduced lumbar lordosis^(37,48,49)^. Barrey et al.^(37)^ evaluated 57 patients with disc degeneration or herniation prior to arthrodesis, in comparison with 154 control subjects, and found the pelvic incidences to be similar between the two groups, although the former group showed lower values for sacral slope, lumbar lordosis, and thoracic kyphosis, as well as higher pelvic tilt values. Rajnics et al.^(49)^ also observed significant differences between patients with herniated disc-related lumbar pain (n = 50) and healthy individuals (n = 30) in terms of the sacral slope, pelvic tilt, and lumbar lordosis parameters. The authors suggested that the lower sacral slope, higher pelvic tilt, and lower lumbar lordosis observed in the patients would lead to an increase in the compression forces applied to the anterior components (vertebral bodies and intervertebral discs), contributing to disc degeneration.

A recently introduced biplane X-ray imaging system (EOS; EOS Imaging, Paris, France) allows the simultaneous acquisition of full-body biplane projections (anteroposterior and lateral) with a radiation dose significantly lower than that of a single radiography, providing a complete evaluation of the spinal deformity and revealing any compensatory mechanisms recruited by the patient^(46,50)^. However, this technique is not yet widely available.

The measurement of multiple spinopelvic parameters offers a more complete view of the relationship between the deformity and its compensation. However, the assessment with multiple parameters can be complicated and requires time and experience. The use of a single parameter that combines anteversion of the trunk and the pelvic tilt tends to be a good option for the screening of possible deformities of the sagittal alignment. Future studies comparing the global tilt, spinopelvic angle, and T1 pelvic angle parameters should be encouraged.

## CONCLUSION

We have presented a review of the spinopelvic parameters that are most relevant in clinical practice. A better understanding of the interaction between the spine and the pelvis, as well as of the compensatory mechanisms involved in the presence of deformities, allow the appropriate diagnosis and a more sophisticated approach to managing the care of patients with spinal deformities.
